# Supplementary material for: Reticuline and Coclaurine Exhibit Vitamin D Receptor-Dependent Anticancer and Pro-Apoptotic Activities in the Colorectal Cancer Cell Line HCT116
Source: Curr Issues Mol Biol. 2025 Oct 1;47(10):810. doi: 10.3390/cimb47100810 (PMC12564130; doi:10.3390/cimb47100810)
Supplement: Supplementary file 1 [file cimb-47-00810-s001.zip › cimb-3878374 supplementary.pdf]

Supplementary Figures

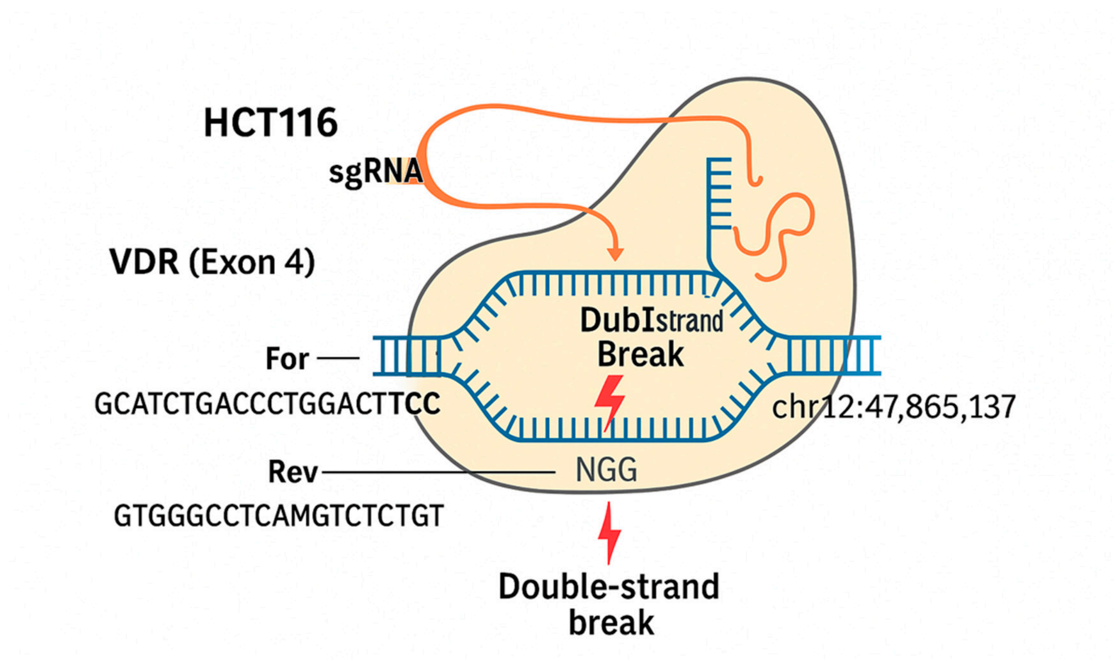

Supplementary Figure S1. Methods generating CRISPR/Cas9-mediated VDR knockout CRC cell line HCT116. Schematic representation of the CRISPR/Cas9 gene-editing methodology employed to produce VDR/KO in HCT116 cells. The guide RNA (gRNA) specifically targets the VDR gene located at chr12:47,865,137. The gRNA and tracrRNA assemble with Cas9, guiding the ribonucleoprotein (RNP) to create a site-specific double-strand break adjacent to the protospacer adjacent motif (PAM) region within the VDR locus.

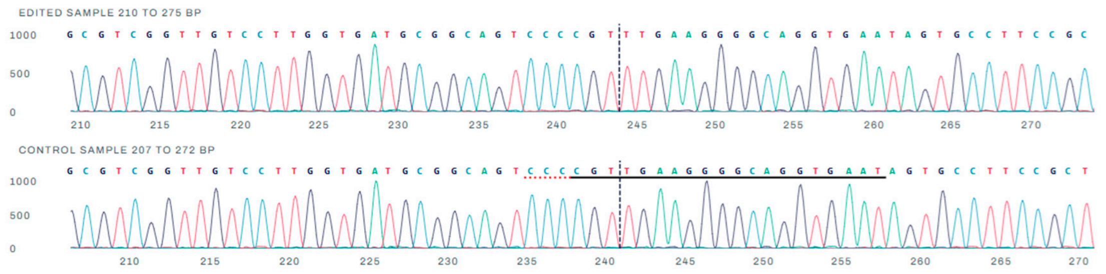

Supplementary Figure S2. The Sanger sequence view showing VDR (edited) and WT (control) sequences in the region around the guide sequence. Sanger sequencing chromatograms validating CRISPR/Cas9-mediated VDR gene editing. The upper panel displays the edited HCT116-VDR/KO sequence, exhibiting significant insertions and deletions next to the CRISPR cleavage site. The lower panel presents the unaltered WT sequence for reference. The dashed line indicates the predicted Cas9 cleavage location.

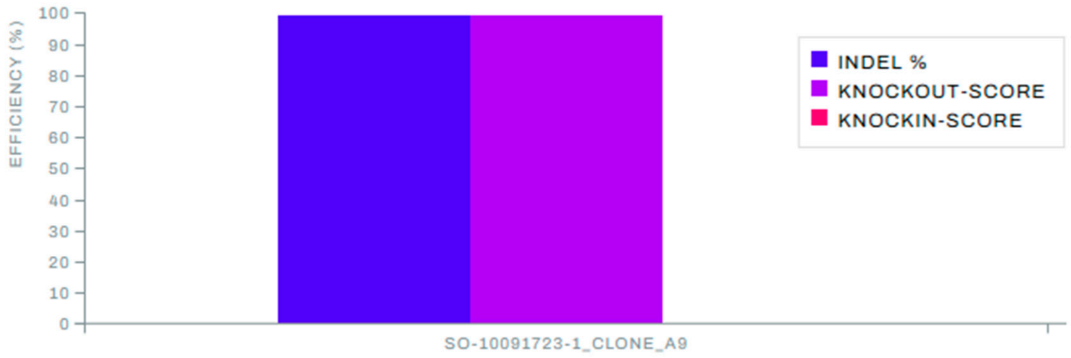

Supplementary Figure S3. CRISPR editing efficiency of VDR-KO. This bar graph shows that 99% of the cells with contain the desired mutation with 99 % of INDL of VDR gene after CRISPR editing.
